# Determinants and Outcomes of Emergency Caesarean Section following Failed Instrumental Delivery: 5-Year Observational Review at a Tertiary Referral Centre in London

**DOI:** 10.1155/2015/627810

**Published:** 2015-05-11

**Authors:** Sian McDonnell, Edwin Chandraharan

**Affiliations:** St. George's University Hospitals NHS Foundation Trust, Blackshaw Road, London SW 17 0RE, UK

## Abstract

*Objectives*. To review the determinants for a failed operative vaginal delivery and to examine associated fetal and maternal morbidity. *Design*. Retrospective observational study. *Setting*. Large London Teaching Hospital. *Method*. A retrospective review of case notes during a 5-year period was carried out. *Results*. Overall 119 women (0.44%) out of 26,856 births had a caesarean section following a failed instrumental delivery, which comprised 5.1% of all operative vaginal births. 73% had a spontaneous onset of labour and 63% required syntocinon at some time prior to delivery. 71.5% of deliveries were complicated by malposition. Only 20% of deliveries were attended by a consultant obstetrician. Almost 50% of women and 8.4% of neonates sustained trauma at the time of either their failed instrumental delivery or the caesarean section. *Conclusions*. Emergency caesarean section during the second stage of labour is associated with maternal and fetal complications. A ‘failed instrumental delivery score' (FIDS) may aid practitioners in predicting an increased likelihood of a failed operative vaginal birth and therefore to consider a trial of operative vaginal delivery in the theatre. Senior input should also be sought because a failed operative vaginal birth is associated with increased maternal and fetal morbidity.

## 1. Introduction

Caesarean section in the second stage of labour is a technically difficult procedure, especially when performed after an operative vaginal delivery has been attempted and when the fetal head is deeply impacted within the pelvis. Therefore, a “second stage” caesarean section may be associated with increased maternal and fetal morbidity [1–4]. Although operative vaginal births are also associated with fetal trauma [5, 6], significant maternal and fetal trauma can also occur during a caesarean section that is performed during late second stage of labour. The rising rates of caesarean section at full dilatation not only are a concern for the delivery in question but also may have a negative impact on woman's future pregnancies and deliveries [7].

A recent 10-year study of operative delivery in a large London teaching hospital has shown a trend to choose a ventouse (vacuum extractor) over forceps and opting for delivery in the operating theatre as well as a small increase in the rate of caesarean section at full dilatation [8]. This study also showed an increase in failed instrumental delivery (correlation coefficient 0.93, *p* < 0.05) which was thought to be due to both instrument failure and a reluctance to attempt instrumentation during second stage of labour.

Other studies have also noted the rise in numbers of caesarean sections at full dilatation [9, 10] and both the Royal College of Obstetricians and Gynaecologists [11] and the American College of Obstetricians and Gynaecologists [12] have advocated the need for further training on instrumental vaginal deliveries.

The aim of this study is to review the determinants for a failed operative vaginal delivery and thereby emergency caesarean sections at full dilatation as well as to determine associated fetal and maternal morbidity.

## 2. Methods

All women who delivered by caesarean section after a failed instrumental delivery at St. Georges Hospital, London, between July 2007 and June 2012, were identified. This London teaching hospital has over 5000 deliveries a year, with three tiers of obstetricians (registrar ST3-5, senior registrar ST6-7, and consultant) working on labour ward. There was always at least the registrar plus senior registrar or consultant on site 24 hours a day, seven days a week. All of the women whose case notes were obtained were over 37 weeks of gestation and had a cephalic presentation.

A proforma was created and completed from the case notes of each woman, detailing background characteristics as well as details surrounding the labour and delivery. Maternal complications that were considered were haemorrhage, intraoperative complications, and genital tract trauma. Neonatal morbidity included Apgar scores, cord arterial pH, and evidence of scalp or fetal lacerations and cephalhematoma.

Information regarding the use of instruments, the total number of instruments (with different types of forceps being classed as two separate instruments), the number of pulls with each instrument during the delivery, and the number of times the cup detached from the fetal head was also recorded. All ventouse deliveries at St. Georges Hospital are performed using the Kiwi Omnicup and metal and silastic cups are not used.

This study was deemed exempt from the need for ethical approval as it is a retrospective observational analysis performed by review of case notes with no clinical interventions and with results showing no identifiable patient data.

## 3. Results 

A total of 119 women from a cohort of 26,856 deliveries required a caesarean section (0.44%) after failed operative delivery. This is compared to 3881 successful operative vaginal deliveries over this time. Our overall failed instrumental delivery rate (total number of failed instrumental deliveries/total number of instrumental deliveries) was 5.1%.

Case notes were obtained for a total of 119 women. Of these 119 women, 22 were delivered for CTG (cardiotocograph) abnormalities and the other 97 because of failure to progress in the second stage of labour.

### 3.1. Determinants of Failed Instrumental Delivery

105 women were primiparous and 14 were multiparous. Of these 14 multiparous women, only one woman had had two previous deliveries. The other 13 women had only one previous delivery; therefore in total there had been 15 previous deliveries.

With respect to their previous deliveries, 5 women had had a previous caesarean section at ≥8 cm, 2 had an elective caesarean section for breech, 4 had a spontaneous vaginal delivery, and 4 had required an operative vaginal delivery during their previous labour. Characteristics of women who had a failed instrumental vaginal delivery (FID) are given in Table 1.

### 3.2. Adverse Outcomes

25% of women in our study had a postpartum haemorrhage (Table 2) and almost half of all women sustained maternal trauma at the time of the attempted operative vaginal delivery or caesarean section (Table 3).

Overall, 8.4% of neonates sustained trauma (Table 4) following FID. 40 out of 106 neonates had a low Apgar score or an umbilical cord arterial pH of < 7.1 (Table 5). In 13 cases (10.9%), cord blood gases were not available.

## 4. Discussion

To the best of our knowledge, our study is the largest study that analyzed the determinants as well as maternal and fetal outcomes for emergency caesarean sections performed for FID over a 5-year period. A number of studies have previously looked at predictors of failed operative vaginal delivery [1, 3, 13] and have concluded that risk factors for FID included

- persistent OP presentation;
- birthweight > 4 kg;
- maternal body mass index >30;
- mid-cavity delivery or when 1/5th of the fetal head is palpable per abdomen.

Murphy et al. [1] also concluded that instrumental delivery, whether successful or not, was associated with increased risk of maternal trauma and increased neonatal trauma (if there were >3 pulls). Multiple instrument usage was associated with increased neonatal trauma as well as initial attempt at vaginal delivery by an inexperienced operator.

Considering previous deliveries in multiparous women, Hoskins and Gomez [14] in 1997 found that having a previous caesarean section at full dilatation reduced the chance of a successful subsequent vaginal delivery to 13%. This is compared to a success rate of 73% and 67%, respectively, if their previous caesarean section was at 6–9 cm or 5 cm or less.

Malposition was a key factor in our cohort of women who had a failed instrumental delivery. In only 29% of women was the fetal head in a direct, right, or left occipitoanterior position. There are no randomized control trials looking at the optimal method of delivery when there is malposition. Options include manual rotation and direct traction forceps, rotational vacuum extractor, or Keilland forceps and each of these options has its own relative merits and demerits. However, Keilland forceps require additional expertise because of the additional risks they confer. Therefore, in our unit, only those who can demonstrate competency and regularly perform Keilland forceps delivery are permitted to do so. Tempest et al. [15] suggest that women are more likely to need a caesarean section if rotational ventouse rather than Keilland forceps is used to assist the birth (OR 8.2; 95%CI 4.54–14.79) and the adverse maternal and neonatal outcomes are comparable when delivery is by Keilland forceps compared to failed rotational ventouse and subsequent caesarean section.

In our unit, the Kiwi Omnicup is the recommended instrument for rotational deliveries. It was chosen as the first instrument in 91 of the 119 cases (76%) with 36 (40%) of them having a second instrument (nonrotational or rotational forceps) applied. In 2001 Vacca reported a 98% success rate [16] for the Kiwi Omnicup in his cohort which included 18 nonrotational and 32 rotational deliveries. However, more recent randomized control trials in the United Kingdom concluded that the Kiwi Omnicup was less successful at achieving a successful vaginal delivery when compared to a “standard” cup (34% versus 21%) and thereby increases the rates of sequential instrument use [17]. However, operator experience and skill need to be considered whilst interpreting the data. Whether the use of the Kiwi cup rather than other rotational instruments is a factor for the failed instrumental rate cannot be determined from our data as this comparison could not be made.

From our data, it can also be seen that failed instrumental deliveries are more common out-of-hours with 60% occurring between 2001 and 0759. Whilst it is not possible to conclude that lack of competency and experience contributed to failed instrumental births, instrumental deliveries are predominantly undertaken by trainees during out-of-hours. Lack of consultant presence on labour ward during out-of-hours has been an issue which the Royal College of Obstetrics and Gynaecology has been attempting to address over recent years [18].

In our study, a large proportion of trials of instrumental delivery were by trainees, although most of these were by obstetricians with over 5-year experience. The impact of the shortening of obstetric training within the UK as a result of the European Working Time Directive may have resulted in trainees being less skilled and consequently having a higher failure rate of instrumental deliveries compared to their consultant colleagues.

Of the women that required syntocinon, 35% commenced syntocinon in the later stages of labour (at or more than 8 cm). This illustrates the importance of carefully assessing the causes of “secondary arrest” of labour and having senior input if instrumental vaginal delivery is subsequently required in these cases.

More than 80% of women also had a second stage lasting for more than four hours. The National Institute for Clinical Excellence Intrapartum Guidelines [19] stated that after 2 hours of active pushing, primiparous women should have a diagnosis of “delay” made (i.e., failure to progress) and plans should be put in place for an operative delivery to occur enabling primiparous women to be delivered within 3 hours of the active second stage starting. This illustrates the importance of having definite endpoints in the second stage of labour and to strike the right balance between promoting normality and reducing the risks of a prolonged second stage of labour.

The station of the fetal head may also be a determinant of failed instrumental delivery. According to the Royal College of Obstetrics and Gynaecology [11], mid-cavity delivery is defined as when the leading point of the fetal skull is above station plus 2 cm but not above the ischial spine. Just over 95% of our cases are therefore defined as mid-cavity and therefore, should be performed by an experienced operator because of the need for a high level of clinical and technical skill.

Body mass index of over 30 is generally thought to be a risk factor for failed instrumental delivery although this was not borne out in our analysis.

Fetal factors that contribute to a failed instrumental delivery are difficult to be predicted, both antenatally and during the intrapartum period. For example, a fetal weight of more than 4 kg is associated with increased likelihood of failed instrumental delivery but there is no good evidence to support the use of ultrasound for estimation of fetal weight due to its inaccuracy [20]. Clinical skills therefore remain important in the diagnosis and management of failure to progress in second stage. It has been reported [20] that clinical examination was found to be significantly more likely within 10% of the actual weight than an ultrasound derived estimation of fetal weight (58% versus 32%; RR 1.65; 95% CI 1.42–1.69). It is therefore unlikely that fetal factors such as weight could be used to predict the likelihood of either successful or failed instrumental delivery.

When considering maternal outcomes associated with FID, approximately 25% of women in our study lost more than 1000 mL at the time of their caesarean section. In the study by Murphy et al. [3], only 10% of women lost more than 1000 mL at the time of their caesarean section but this was significantly more than those women who achieved a vaginal delivery (adjusted OR 2.8, 95% CI 1.1–7.6). Their group also showed that increased blood loss was less likely with an experienced obstetrician but in our cohort that did not appear to be the case. This increase in blood loss with a fully dilated caesarean section as compared to vaginal delivery was also noted by Ebulue et al. in 2008 [21] (802.7 ± 100.0 versus 425.4 ± 120.0 mL). We run regular “fire drills” on estimation of blood loss in our unit for all staff and therefore it is very likely that the higher EBL noted in our study reflects a more accurate estimation of blood loss at delivery. In addition, obstetric trainees were involved in delivery of 80% of cases who sustained a postpartum haemorrhage of > 1000 mL (Table 2).

Maternal trauma sustained at the time of delivery can occur either at the time of attempted vaginal delivery or during the emergency caesarean section following FID. In our study, a total of 66 “episodes” of maternal trauma were documented. Eight women sustained trauma via two separate mechanisms whereas 61 women did not sustain any trauma at the time of delivery. Therefore, 48% of women sustained trauma at the time of their failed instrumental vaginal delivery or caesarean section. Over 25% of the women who sustained trauma had vaginal/perineal injuries. There is no evidence to support the routine use of episiotomy at the time of operative vaginal delivery [11]. Macleod and Murphy [22] surveyed practicing obstetricians with regard to operative delivery and the use of episiotomy. They found that a restrictive approach was preferred for deliveries using a ventouse (72%) but a routine approach for forceps (73%). Even with such an approach, episiotomies should not be performed until the stage where delivery is deemed to be imminent. Therefore, it is essential to avoid an episiotomy when the fetal head is at station 0 or plus 1 cm when there is minimal or no descent with traction, to avoid an inappropriate episiotomy.

Our study highlights the fact that both the incidence and severity of maternal trauma are greater when an emergency caesarean was performed for FID, where the primary indication was failure to progress in labour. Therefore, optimization of management of second stage of labour and providing experienced obstetric input is paramount to avoid these complications.

Neonatal outcomes at the time of failed operative delivery and subsequent caesarean have been considered by a number of studies in the past. Unfortunately, it is difficult to compare our data with these studies due to a wide variation in neonatal complications (neonatal unit admissions, jaundice, sepsis, and seizures) that have been considered by individual studies. Much of the available evidence suggests that sequential instrumentation should be avoided if possible because of the increased neonatal morbidity [1]. Murphy et al. found that the use of sequential instruments was associated with increased neonatal trauma (adjusted OR 3.1, 95% CI 1.5–6.8 and adjusted OR 4.4, 95% CI 1.3–14.4, for completed and failed deliveries, resp.). In our study, 34 women (29%) had sequential instruments with either ventouse and forceps or nonrotational and rotational forceps. In half of these cases, there was malposition of the fetal head. Loss of scalp tissue and laceration of the eye (Table 4) highlight operator factors and the need to determine the fetal position accurately, if necessary, using an ultrasound scan to identify fetal orbits, to avoid these complications.

## 5. Conclusion

Emergency caesarean section during second stage of labour is associated with maternal and fetal complications and also has the potential to negatively influence a woman's birth experience. Our study has shown that failed instrumental delivery is more likely with fetal malposition, prolonged second stage of labour, use of oxytocin for secondary arrest, and lack of operator experience. It is also associated with maternal and neonatal morbidity. Although current guidelines on operative vaginal delivery do identify “risk factors” that may increase the incidence of failed instrumental delivery, there are no scoring systems to aid obstetricians in determining the likelihood of failure. Based on the findings of our study that analyzed emergency caesarean sections for FID in 119 women, we have formulated a Failed Instrumental Delivery Score to aid clinicians on the “shop floor” in determining the likelihood of failure (Table 6). We have suggested that if the Failed Instrumental Delivery Score is ≥ 8, there is an increased likelihood of a failed instrumental vaginal birth and hence a trial of instrumental vaginal delivery in the theatre should be considered and the consultant on call should be alerted in view of associated increased maternal and fetal morbidity due to FID. We sincerely hope that use of such clinical scoring system based on key parameters that could be easily determined prior to attempting an instrumental vaginal delivery would help clinicians to ensure availability of an experienced clinician and also to conduct delivery in an appropriate environment with a ready recourse to caesarean section. A larger prospective trial may help in confirming the usefulness of the FID Score.

## Figures and Tables

**Table 1:** Characteristics of women who had a failed instrumental vaginal delivery.

Characteristics		
Body mass index	>30 Kg/m^2^	10 (8.4%)

Onset of labour	Spontaneous	87 (73.1%)
	Induced	21 (17.6%)
	Augmented	11 (9.2%)

Use of oxytocin	None	44 (37.0%)
	Yes	75 (63.0%)
	<4 cm = 19, 4–7 cm = 30, 8–10 cm = 26	

Position of fetal head	Right/left/direct occipitoanterior	34 (28.5%)
	Right//left/direct occipitoposterior	40 (33.6%)
	Occipitotransverse	43 (36.1%)
	Others	2 (1.8%)

Station of the fetal head (distance of the leading bony point of fetal skull below the ischial spines, measured in centimeters)	Above −1	1
	−1	2
	0	68
	+1	48
	+2	2

Fetal size	Mean 3588 g (2365 g–4840 g)	

Operator experience	Trainee <5 years	13 (10.9%)
	Trainee 6-7 years	82 (68.9%)
	Consultant (>8 years)	24 (20.2%)

Time of decision to perform operative vaginal birth	0800–1700	36 (30.3%)
	1701–2000	11 (9.2%)
	2001–0759	72 (60.5%)

Length of second stage of labour (in cases where the indication was “failure to progress”)	<3 hr	5.2%
	3.01–≤4 hr	14.4%
	≥4.01 hr	80.4%

**Table 2:** Maternal outcomes: postpartum haemorrhage (%).

Estimated blood loss	Total	Trainee (<5 years)	Trainee (6 or 7 years)	Consultant (<8 years)
<500	19 (16.0%)	2 (18.2)	13 (15.4)	4 (16.7)
500–999 ml	69 (58.0%)	7 (63.6)	48 (57.1)	14 (58.3)
1000–1999 ml	30 (25.2%)	2 (18.2)	22 (26.2)	6 (25.0)
≥2000 ml	1 (0.8%)	0	1 (1.2)	0

**Table 3:** Maternal outcomes: birth trauma.

Complication	Indication: abnormal CTG (%)	Indication: failure to progress (%)
Nil	11 (50)	50 (48.5)
Episiotomy	2 (9.1)	2 (2.1)
Perineal tear/graze	1 (4.5)	12 (12.8)
Uterine extension	8 (36.4)	30 (30.9)
Delivered as breech	2 (9.1)	2 (2.1)
Broad ligament haematoma	0	3 (3.1)
Wound dehiscence	0	1 (1.0)
Inverted T	0	1 (1.0)
Bladder injury	0	1 (1.0)
Urethral tear	0	1 (1.0)

**Table 4:** Neonatal outcomes: trauma.

Trauma	Number (out of a total of 119 babies)
Scalp loss	5
Laceration over eye	3
NNU admission	1
Neonatal death (sepsis)	1
Total	10 (8.4%)

**Table 5:** Neonatal condition at birth: Apgar scores and umbilical cord arterial pH.

	Number (1 unable to obtain; umbilical cord gases were not documented in 12 cases)
Arterial pH <7.1	14/106
Apgar <7 at 1 min	20/106
Apgar <7 at 5 min	6/106

**Table 6:** Failed Instrumental Delivery Score.

	0	1	2
Position of presenting part	ROA/LOA/DOA	ROP/LOP/DOP	Others
Commencement of oxytocin at dilatation	≤4 cm	5–7 cm	≥8 cm
Duration of second stage (hrs)	<3	3-4	>4
Experience of the operator (years)	>8	6-7	≤5
Parity	≥3	1-2	<1

## Abbreviation

FID: Failed instrumental delivery.

## Key Message

A proportion of emergency caesarean sections following failed instrumental deliveries may be potentially avoidable. An intrapartum clinical scoring system to determine the adverse factors that are associated with FID may help clinicians to optimize management of second stage of labour.
